# Evaluation of Coronary Artery Calcification by Multi-detector Row Computed Tomography for the Detection of Coronary Artery Stenosis in Japanese Patients

**DOI:** 10.2188/jea.15.187

**Published:** 2005-09-27

**Authors:** Akihiko Kitamura, Tohru Kobayashi, Kouki Ueda, Takeo Okada, Nobuhisa Awata, Shinichi Sato, Takashi Shimamoto

**Affiliations:** 1 Osaka Medical Center for Health Science and Promotion.; 2 Osaka Medical Center for Cancer and Cardiovascular Diseases.

**Keywords:** Calcinosis, Coronary Disease, Arteriosclerosis, Tomography, X-Ray Computed, Patients

## Abstract

BACKGROUND: The development of an efficient noninvasive examination to detect coronary atherosclerosis is needed as a strategy to prevent coronary heart disease. To evaluate the usefulness of calcium score measured by multi-detector row computed tomography (MDCT), we compared calcium score derived from MDCT with findings of coronary artery stenosis assessed by coronary angiography (CAG).

METHODS: In 108 patients (94 men, 14 women; average age, 65.7 years), we performed unenhanced CT scans and calculated coronary artery calcium score in 259 vessels without previous intervention and severe motion artifact to determine the correlation with the degree of coronary stenosis by CAG.

RESULTS: The sensitivity and the specificity of calcification (calcium score 0.1+) for severe stenosis (75+%) were 89% and 43%, respectively. All four vessels with calcium score 1000+ had a severe stenosis. The areas under the receiver operating characteristics curve of calcium score for severe stenosis were 0.80 ± 0.04, indicating the efficacy of this technique.

CONCLUSIONS: Coronary artery calcification and calcium score determined by MDCT were associated with coronary arteries with severe stenosis. This technique appears to be useful for the evaluation of coronary atherosclerosis.

Coronary heart disease has been the leading cause of death in the most countries.^1^ In Japan, we found that that the incidence of ischemic heart disease and associated risk factors, such as serum total cholesterol and body mass index levels, increased from the 1960s to the 1990s among male workers and local residents in an urban area, Osaka.^2^^,^^3^

As a strategy to prevent coronary heart disease, the development of an efficient noninvasive examination to detect coronary atherosclerosis is needed. Techniques to measure coronary artery calcification as a marker of coronary arteriosclerosis using electron-beam computed tomography (EBCT) have been developed and several studies have clarified the association between coronary artery calcification and angiographically significant stenosis.^4^^-^^14^ However, there have been few such studies using multi-detector row computed tomography (MDCT), which has recently come into worldwide use. MDCT is widespread among many general hospitals and examination centers because of its capability of wide application for other parts of body besides the heart. Therefore, high risk patients will have more opportunities to undergo coronary calcium scoring by MDCT as well as by EBCT, if the validity of this technique is confirmed. Several studies have showed a high correlation between calcium scores obtained with MDCT and those obtained with EBCT.^15^^-^^19^

The purpose of this study is to evaluate the efficacy of coronary calcium scoring by MDCT in the detection of coronary atherosclerosis. For this purpose, we determined the sensitivity and specificity of calcium score for substantial coronary artery stenosis assessed by coronary angiography (CAG).

## METHODS

### Subjects

One hundred and eight patients (94 men, 14 women; age range, 48 to 78 years; mean age, 65.7 years) who had ascertained or suspected coronary heart disease underwent CAG and unenhanced computed tomography (CT) examination. Seventy-eight of the 108 subjects had previously undergone percutaneous coronary interventions or coronary artery bypass graft surgery. Of the 108 patients, 59 (55%) had been on antihypertensive medication, 48 (44%) had taken a lipid-lowering medication, and 22 (20%) had taken a medication for diabetes. Current smokers and obesity (BMI 25+ kg/m^2^) accounted for 46% and 41% of respective 93 and 98 patients whose data were available.

### CT protocol

Unenhanced CT of the heart was performed on the day before CAG. CT images were obtained using a 4-row multislice CT system (Aquilion, Toshiba, Tokyo, Japan). The coronary arteries were scanned, using prospective ECG-gated axial scanning with a slice thickness of 3 mm, over a range of 108 mm starting from a point 1.5 cm below the tracheal bifurcation. The scan parameters were 120 kV, 200 mA, 0.32 s (0.5 s half scan). Scanning time was 15-20 seconds, for which time all patients were able to hold their breath. The effective dose with this technique was estimated as 4.27 mGy by computed tomography dose index, which was slightly higher than that with EBCT, which was reported 3.67mGy in a previous study.^16^

### Image analysis

Quantification of calcification was performed on a NetraMD™ workstation with scoring software (ScImage Inc., Los Altos, CA). A calcified lesion in a coronary artery was defined as an area of 0.52 mm^2^ or greater (more than 2 pixels) with CT numbers above a threshold of 130 HU. The region of interest was placed by a single experienced physician (A.K.) around all lesions in the coronary arteries. Measurements of lesion areas and the maximal CT number in each lesion were determined automatically. Then, calcium score in each lesion was computed by the Agatston method.^4^ The Agatston score represented the area score multiplied by an attenuation factor. The attenuation factor was determined based on maximal CT number of the lesion as follows: factor 1 = 130-199 HU, factor 2 = 200-299 HU, factor 3 = 300-399 HU, and factor 4 = 400 HU or greater.

Selective CAG was performed via a radial approach. Angiograms were documented as digital images and evaluated by a panel of experienced cardiologists who had no knowledge of the CT findings. Severe and moderate stenosis was defined as lumen narrowing of 75+ % and 50+ %, respectively.

### Data and statistical analysis

Of the total of 432 vessels, we excluded 118 vessels that had been treated with percutaneous coronary intervention or coronary artery bypass graft surgery, as well as 55 vessels in which it was difficult to calculate the calcium score because of cardiac motion artifacts (Table 1). Motion artifact was more likely to occur in the right coronary artery (RCA), less likely in the left main coronary artery (LM), and intermediately likely in the left anterior descending coronary artery (LAD) and left circumflex coronary artery (LCX). Thus, 259 vessels were analyzed in the present study.

**Table 1.** Number of vessels for analysis.

	

	Total	Coronary interventions	With motion artifacts*	Available for analysis
Right coronary artery (RCA)	108	39	26 (38)	43
Left main coronary artery (LM)	108	4	5 (5)	99
Left anterior descending coronary artery (LAD)	108	48	13 (22)	47
Left circumflex coronary artery (LCX)	108	27	11 (14)	70

Total	432	118	55 (18)	259

Numbers in parentheses represent proportions calculated as follow: number of motion artifact / (total number - number of interventions) ×100.

*: Calcium score was not available because of cardiac motion artifact.

The sensitivity, specificity, positive predictive value (PPV), and negative predictive value (NPV) of calcium score for severe and moderate stenosis were calculated with 2×2 contingency tables. Receiver operating characteristic (ROC) curve analysis^20^ was used as an extension of traditional sensitivity and specificity analyses to establish relationships between calcium score and coronary artery stenosis. For ROC analysis, logistic regression analysis was used to calculate age-adjusted sensitivity and specificity and we used a logarithmic transformation of the calcium score +1 because of the skewed distribution of the calcium score.

The study protocol was approved by the Human Ethics Review Committee of Osaka Medical Center for Cancer and Cardiovascular Diseases and informed consent was obtained from each patient.

## RESULTS

The sensitivity of calcification (calcium score = 0.1 or greater) in severe stenosis was 89%, and specificity, PPV, and NPV were 43%, 20%, and 96%, respectively (Table 2). The sensitivity and NPV in moderate stenosis were lower, while specificity and PPV were higher as compared to those in severe stenosis.

**Table 2.** Sensitivity, specificity, and predictive values of calcification for the detection of severe and moderate stenosis.

	Severe stenosis		Moderate stenosis	
Sensitivity (%)	89	(32/36)	84	(59/70)
Specificity (%)	43	(96/223)	47	(89/189)
Positive predictive value (%)	20	(32/159)	37	(59/159)
Negative predictive value (%)	96	(96/100)	89	(89/100)

Table 3 shows the sensitivity, specificity, PPV and NPV with regard to severe stenosis in each vessel. In this data analysis, the LM and LAD were pooled together because the area of calcification often extended from the LM to the LAD, with consequent difficulty in discriminating the two areas. The sensitivity and specificity of calcification in the LM and LAD were the highest, while the respective values in the RCA were the lowest.

**Table 3.** Sensitivity, specificity, and predictive values of calcification for the detection of severe stenosis of individual coronary arteries.

	Total		LM+LAD		LCX		RCA	
Sensitivity (%)	89	(32/36)	100	(15/15)	91	(10/11)	70	(7/10)
Specificity (%)	43	(95/223)	47	(62/131)	37	(22/59)	36	(12/33)
Positive predictive value (%)	20	(32/160)	18	(15/84)	21	(10/47)	25	(7/28)
Negative predictive value (%)	96	(95/99)	100	(62/62)	96	(22/23)	80	(12/15)

**Figure 1.**
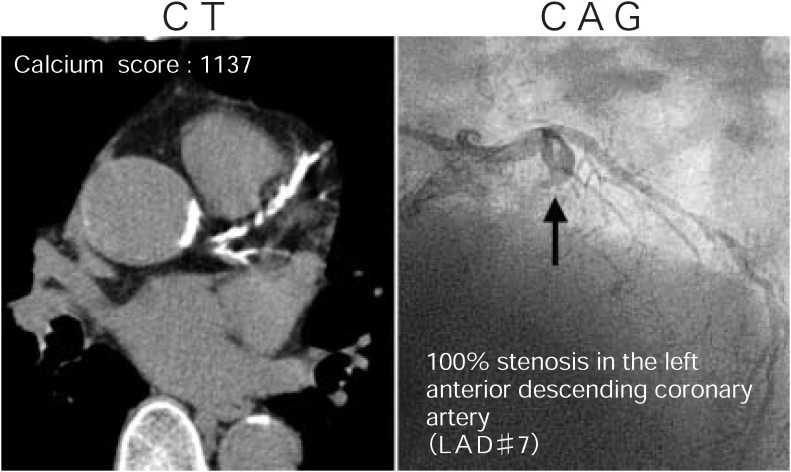
A case of severe calcification (calcium score: 1137) in the LAD in which complete obstruction was demonstrated by CAG.

**Figure 2.**
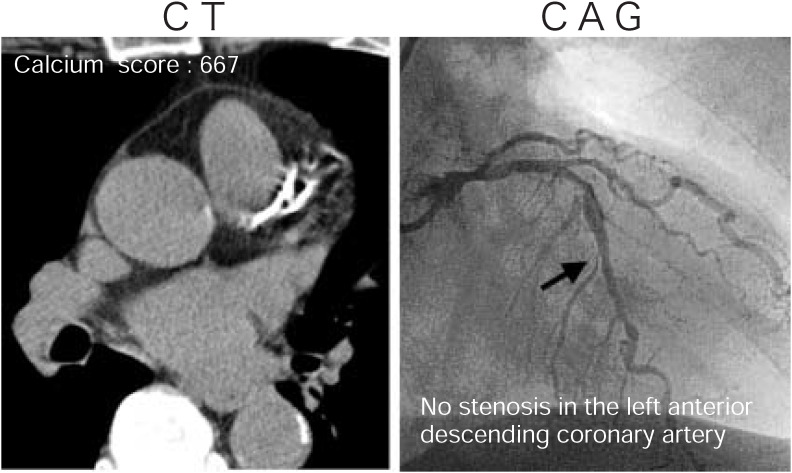
A case of moderate calcification (calcium score: 667) in the LAD in which significant stenosis was not demonstrated by CAG.

**Figure 3.**
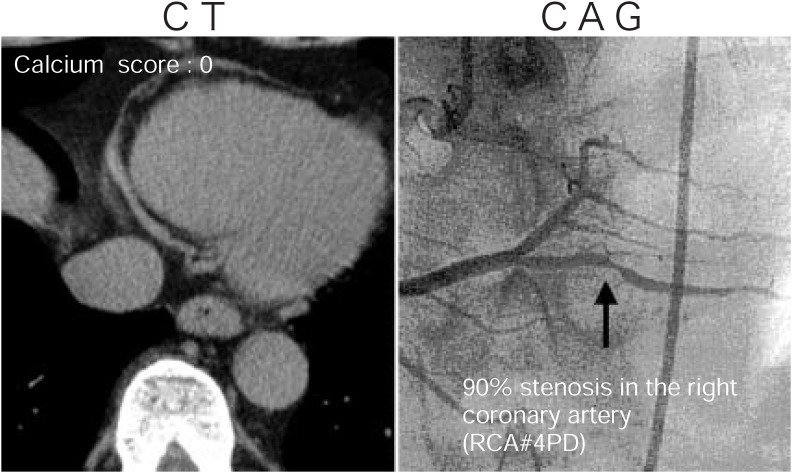
A case without any calcification (calcium score: 0) in the RCA in which severe stenosis (90%) was demonstrated by CAG.

LM: left main coronary artery, LAD: left anterior descending coronary artery, LCX: left circumflex coronary artery, RCA: right coronary artery,

Representative lesions demonstrated by MDCT and CAG are shown in Figures 1 to 3. Figure 1 shows a case with severe calcification in the LAD in which complete obstruction was demonstrated by CAG. A case with moderate calcification in the LAD in which significant stenosis was not demonstrated by CAG is shown in Figure 2. Figure 3 shows a case with no calcification in the RCA in which severe stenosis was demonstrated by CAG.

The sensitivity, specificity, PPV and NPV for severe stenosis according to the calcium score level are shown in Table 4. The sensitivity was lower, and specificity and PPV were higher at higher scores. More than 90% specificity was observed at a calcium score of 300. More than 80% PPV was observed at a calcium score of 900, and PPV was 100% (4 of 4) at a calcium score of 1000+.

**Table 4.** Sensitivity, specificity, and predictive value for the detection of severe stenosis by calcium score level.

Calcium score	Sensitivity (%)		Specificity (%)		Positive predictive value (%)		Negative predictive value (%)	
0.1	89	(32/36)	43	(96/223)	20	(32/159)	96	(96/100)
10	83	(30/36)	56	(124/223)	23	(30/129)	95	(124/130)
50	81	(29/36)	71	(159/223)	31	(29/ 93)	96	(159/166)
100	72	(26/36)	81	(181/223)	38	(26/ 68)	95	(181/191)
200	53	(19/36)	86	(192/223)	38	(19/ 50)	92	(192/209)
300	33	(12/36)	91	(202/223)	36	(12/ 33)	89	(202/226)
400	28	(10/36)	94	(210/223)	43	(10/ 23)	89	(210/236)
500	22	(8/36)	95	(211/223)	40	(8/ 20)	89	(211/239)
800	14	(5/36)	99	(220/223)	63	(5/ 8)	88	(220/251)
900	14	(5/36)	99	(222/223)	83	(5/ 6)	88	(222/253)
1000	11	(4/36)	100	(223/223)	100	(4/ 4)	87	(223/255)

**Figure 4.**
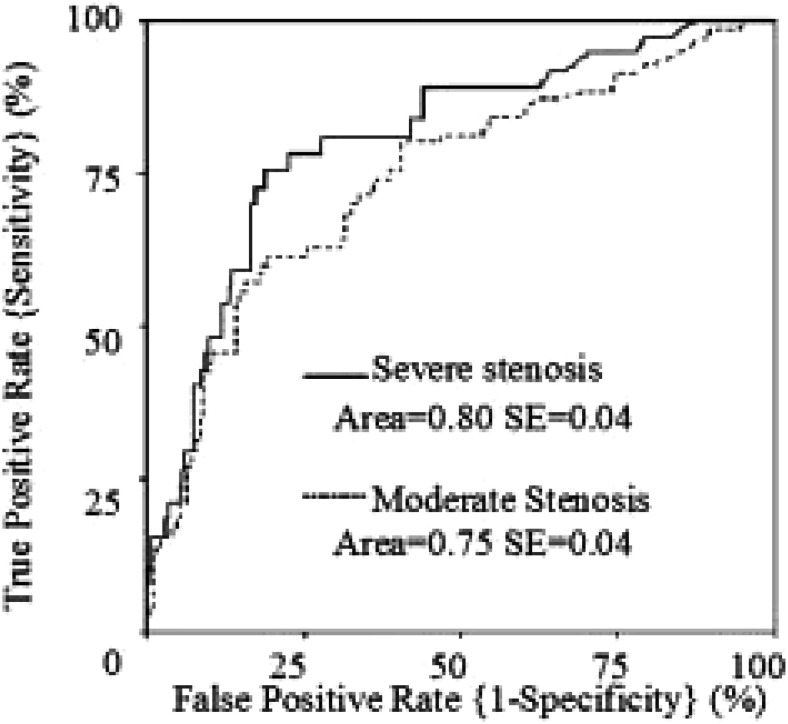
Receiver operating characteristic curves for prediction of stenosis using the calcium score.

The ROC curves for prediction of severe and moderate stenosis using the calcium score are shown in Figure 4. The area (± standard error) under the ROC curve of the calcium score for severe stenosis was 0.80 ± 0.04 (p<0.001), which was greater than the area of 0.75 ± 0.04 (p<0.001) for moderate stenosis.

## DISCUSSION

The major findings of the present study were (1) 89% of vessels with severe stenosis had calcification; (2) 57% of vessels without severe stenosis also had calcification; and (3) all vessels with a calcium score 1000 or greater had severe stenosis.

A recent meta-analysis^14^ of calcification measurement by EBCT showed a pooled sensitivity of 92.3% (95% confidence interval [CI]: 90.7-94.0) and pooled specificity of 51.2% (95% CI: 47.5-54.9) for coronary stenosis 50+ %. From one study of calcification measurement using double-helical CT, the corresponding sensitivity and specificity were 91% and 52%, respectively.^21^ Our findings of calcification for the moderate (50+ %) stenosis group showed 84% sensitivity, which value was slightly lower than that reported in these previous studies. This discrepancy might have been attributable to our analytical method, which was vessel-based rather than patient-based as in the previous studies. Bormann et al.^5^ found that calcium scores were not predictive of a significant stenosis at the calcification site and suggested that the extent and site of calcification does not equate with site-specific stenosis, which means that the presence of calcification in one vessel could suggest a significant stenosis in any part of other vessels. Therefore, patient-based analysis might tend to produce higher sensitivity than vessel-based analysis. However, even in our vessel-based study, sensitivity reached approximately 90% and the area under the ROC curve was 0.80 for severe stenosis (75+ %), which suggested a usefulness of this technique for the evaluation of severe stenosis. Kajinami et al. showed 91%sensitivity and 50% specificity of calcification by EBCT for significant stenoses (75+ % by densitometry),^9^ which were similar to those in the present study.

Limitations of this survey warrant consideration. First, we used the axial CT scanning with a slice thickness of 3 mm (prospective ECG-gated scanning) instead of helical CT scanning (retrospective ECG-gated reconstruction), because of the lower dose of X-ray exposure with the axial scanning than that with helical scanning, which is more important in a noninvasive examination for asymptomatic subjects. However, the axial scanning leaded to 18% of vessels with motion artifacts, which made accurate calcium scoring difficult. Motion artifact is mostly dependent on the duration of scanning. This problem might be improved with advances in multi-detector row systems to scan a wider range simultaneously. At present, a 64 rows multislice CT system is being developed. Second, we examined only the calcification by unenhanced MDCT, although contrast-enhanced MDCT angiography may provide some information on noncalcified plaques and coronary artery walls.^22^^,^^23^ We intend to introduce this non-invasive examination at a examination center as well as a hospital; therefore, a possibility of any side effect with contrast medium should be avoided.

Third, most of the subjects of the present study were male patients with coronary artery diseases and the number of cases was too small to conduct sex-specific and age-specific data analyses like a previous study of Japanese patients with EBCT.^24^ Further studies including more subjects without clinical coronary artery disease, and cost-effectiveness analysis will be necessary to examine the applicability of this technique as a detailed examination for high risk group of coronary heart diseases. We believe that calcium scoring should not been done if patients will be scheduled to undergo coronary catheterization because it just increases the examination cost. We expected the calcium scoring could be used as a diagnostic examination to identify patients who need to undergo coronary catheterization that costs a lot.

In conclusion, coronary artery calcification and calcium score evaluated by MDCT were associated with coronary arteries with severe stenosis in the present study. This noninvasive technique appears to be useful for the evaluation of coronary atherosclerosis.
